# Pneumomediastinum and Pneumothorax as Relevant Complications of Sub-Intensive Care of Patients with COVID-19: Description of a Case Series

**DOI:** 10.3390/medicina57090919

**Published:** 2021-09-01

**Authors:** Maria Gabriella Coppola, Marina Lugarà, Stefania Tamburrini, Pasquale Madonna, Claudio Panico, Giuseppe Noschese, Eduardo Pone

**Affiliations:** 1 UOC Medicina Generale PO Ospedale del Mare ASL Napoli 1 Centro, 80147 Naples, Italy; marinalugara82@gmail.com (M.L.); linomadonna@libero.it (P.M.); 2 UOC Diagnostica per Immagini PO Ospedale del Mare ASL Napoli 1 Centro, 80147 Naples, Italy; tamburrinistefania@gmail.com; 3 UOC Chirurgia Toracica PO Ospedale del Mare ASL Napoli 1 Centro, 80147 Naples, Italy; cl.panico@tiscali.it; 4 Sub Intensiva COVID PO Ospedale del Mare ASL Napoli 1 Centro, 80147 Naples, Italy; giuseppenoschese@gmail.com (G.N.); eddi22@alice.it (E.P.)

**Keywords:** pneumomediastinum, pneumothorax, non-invasive ventilation, COVID-19

## Abstract

Lung failure has been the most common cause of hospitalization for COVID-19. Yet, bilateral interstitial pneumonia has not been the only cause of lung failure of these inpatients, and frequently they develop other illnesses associated with COVID-19. Pulmonary embolism has been the most looked for in the world, but rarely other pneumological diseases, such as pneumothorax and pneumomediastinum, have been described and associated with a worsening prognosis. We here report our clinical experience associated with the occurrence of pneumothorax and pneumomediastinum in a cohort of inpatients hospitalized in our division of medicine in a regular ward or in a sub-intensive ward.

## 1. Background

Pneumomediastinum and pneumothorax are rarely diagnosed in an emergency room [1,2]. Usually, spontaneous pneumomediastinum is more frequently described in young patients with a history of asthma or recent inhalation of recreational drugs [2]. Pneumothorax is classified as primary spontaneous pneumothorax in persons without clinically apparent lung disease-associated risk factors, including male sex, tall stature, smoking and a family history of pneumothorax or secondary spontaneous pneumothorax as a complication of preexisting lung diseases, post-traumatic lung disease or iatrogenic lung disease [1].

Pneumomediastinum and pneumothorax causes include barotrauma in patients’ asthma, COPD, pulmonary infections, bronchiectasis and more (e.g., lung cysts, lung malignancy or iatrogenic injuries from endoscopy, surgery or central line placement) [3].

On the other hand, pneumomediastinum and pneumothorax have been reported as uncommon complications in pneumonia, caused by staphylococcus, interstitial pneumonitis and also including Pneumocystis carinii associated with human immunodeficiency virus (HIV) and/or tuberculosis [4].

During the pandemic caused by SARS CoV2, due to the complexity of the disease and therapeutic approach, an increase of pneumomediastinum and pneumothorax have been reported in the daily clinical management, although the epidemiology of this complication is difficult to be understood due to multiple case reports, few cohorts are present in the literature [5]. 

From a clinical point of view, the occurrence of pneumomediastinum and pneumothorax may induce a severe clinical complication of all acute illnesses (e.g., COVID-19), which may increase the morbidity and mortality of patients.

We have reported a series of six patients with pneumothorax and pneumomediastinum associated with COVID-19 in our cohort of patients.

## 2. Description of Cases

During the SARS-CoV-2 pandemic, in our sub-intensive area of the Medicine-COVID-19 unit, we observed six cases of pneumomediastinum and one case of pneumothorax as a complication of the clinical scenario in patients with SARS-CoV-2 associated pneumonia.

Pneumomediastinum and pneumothorax are also reported as rare complications of NIV, but the real frequency during other medical illnesses is still unknown. Patients that we found affected by pneumomediastinum, in fact, showed progressive lung failure and required therapeutic support with non-invasive ventilation (NIV). The most common complications of NIV are infections by bacteria of the high respiratory tract, as far as for all patients that require mechanical ventilation [6,7].

**Figure 1:**
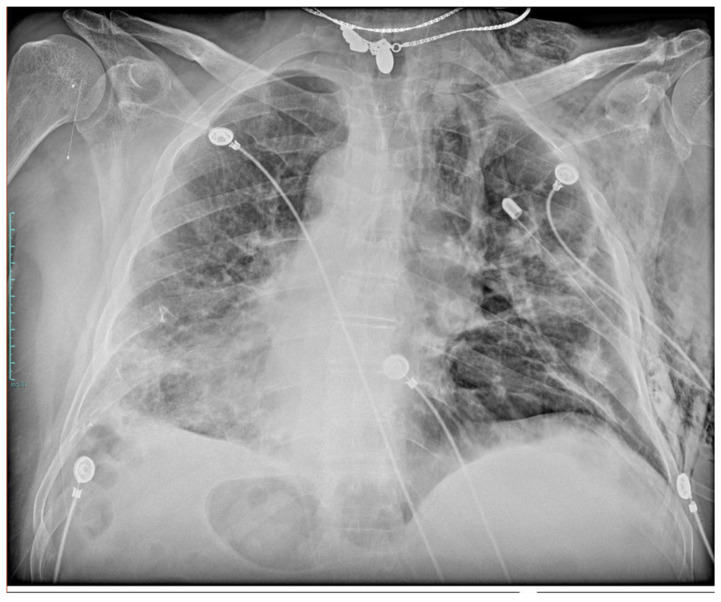
Chest X-ray showing right-sided chest tube placed for pneumothorax and mediastinal emphysema in COVID-19 patient.

**Figure 2:**
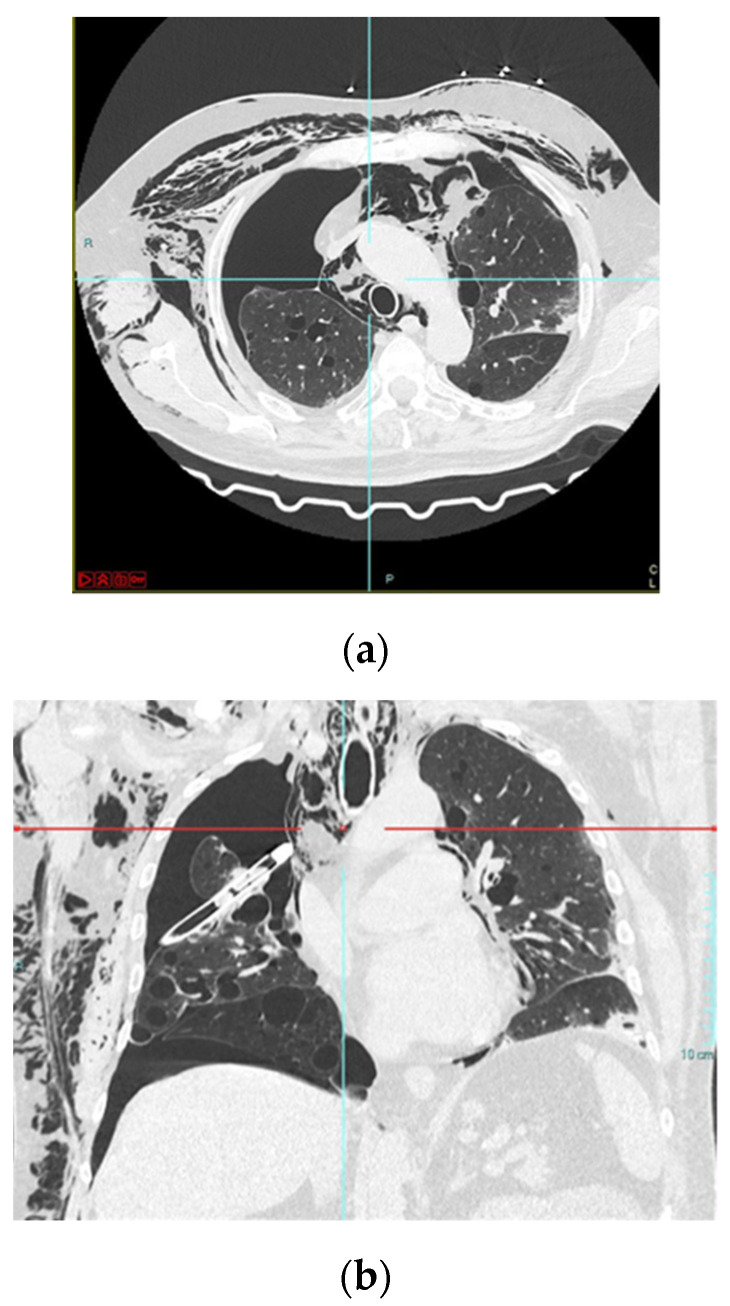
A typical case of a COVID-19 pneumothorax and pneumomediastinum patient (**a**) chest TC showing pneumothorax extended at 75% of the right lung and large mediastinal emphysema; (**b**) Coronal CT showing chest drainage for pneumothorax at right lung anteroposterior projection image.

In our study, we reported the occurrence of pneumothorax and pneumomediastinum during treatment in a sub-intensive area of our division of medicine for all patients affected by lung failure for COVID-19. All patients were males with a mean age of 59.63 years. From a clinical point of view, the younger patients presented with a small spontaneous pneumomediastinum and positivity to nasopharyngeal swab for SARS-CoV-2 while radiological findings of interstitial pneumonia were rarely detected; his personal anamnesis did not reveal smoking or COPD or any type of trauma before hospitalization or recent treatment with NIV for SARS-COV 2 infection. This patient had not complained of any respiratory symptoms. The small pneumomediastinum was only detected incidentally to the chest CT. Another patient with a history of bullous emphysema showed subcutaneous emphysema over the lower neck, spontaneous extensive right-sided tension pneumothorax, small left-sided pneumothorax and pneumomediastinum on chest-X-ray a few days after the positive nasopharyngeal swab to SARS-CoV-2. He went to the emergency room of another hospital with a *sudden breathlessness* crisis, and he was treated with immediate drainage catheter placement (Figure 1 and Figure 2). He was *subsequently transferred* to our COVID-19 Unit. This latter patient had no other comorbidities, rarely took ICS/LABA/LAMA therapy at home and had no previous episodes of pneumothorax.

From a radiological point of view, five of the six patients showed extensive bilateral interstitial pneumonia without associated pulmonary embolism, but three of them showed an overlapping gram-negative aerobic bacterial infection (e.g., Acinetobacter baumanii, Pseudomonas fluorescens or Corynebacterium striatum) requiring multiple rounds of broad-spectrum antibiotics.

The other clinical characteristics and comorbidities of observed patients are summarized in Table 1. 

**Figure 3:**
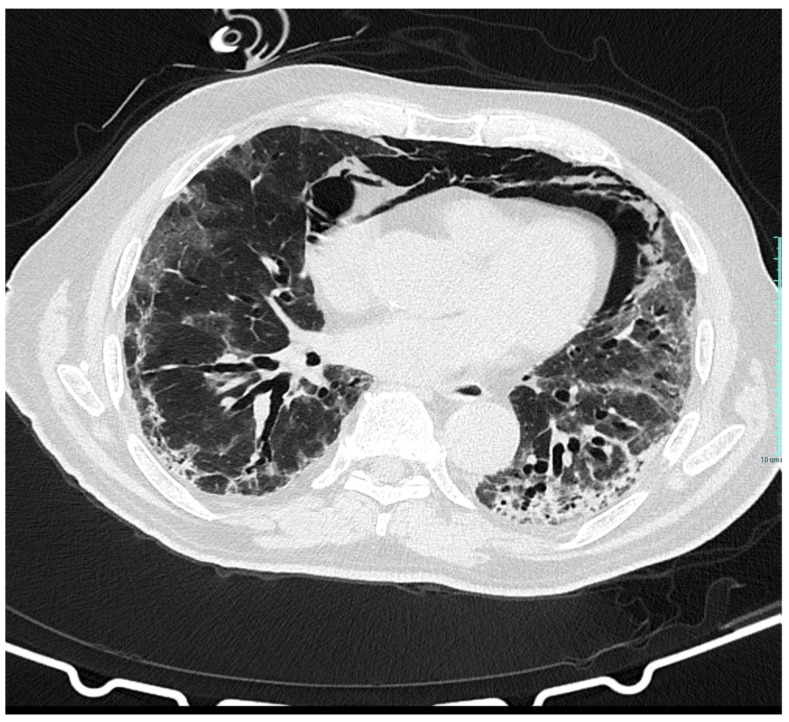
Coronal CT image shows multiple mixed ground-glass opacities in both lungs and pneumomediastinum.

Four patients during the hospital course received non-invasive positive pressure ventilation (NIPPV) with a Pressure Support (PS) of 5–7 cmH2O and a positive end-expiratory pressure PEEP started at 10 cmH2, achieving an average tidal volume of about 450 mL of developed pneumomediastinum. After a good response to NIPPV therapy, PEEP was reduced to 8 cmH2. Yet, patients presented a fluctuating clinical response to therapy and, after significant respiratory distress and a subsequent chest CT, demonstrated an extensive pneumomediastinum. Physical examination showed bilateral cervical subcutaneous emphysema, spreading to both supraclavicular fossae, also confirmed by CT scan (Figure 3).

Regarding patients that received NIPPV, the average number of days between ventilation and subsequent barotrauma was 8.5 days. Four patients did not show personal anamnesis comorbidities associated with the increased risk of pneumomediastinum. Two of them had a medical history significant for hypertension, Type 2 diabetes, and one of them also had ischemic heart disease.

We treated pneumomediastinum in a conservative way in all patients by monitoring via HRCT, where gradual improvement was observed. One of them, because of an underlying ischemic heart disease, died with acute cardio-respiratory failure during hospitalization. Only one patient showing extensive pneumothorax required drainage because of a worsening respiratory condition.

However, the occurrence of these kinds of complications also induced a prolonged average of hospitalization length (mean vs. 24, 28 days).

At our hospital, the incidence of pneumomediastinum/subcutaneous emphysema was 5.45%, and the incidence of pneumothorax was 1.1%. Several clinical cases have been found in the literature describing pneumomediastinum or pneumothorax, or both simultaneously, and related to barotrauma, as complications of COVID-19 infection [5,8,9,10]. One study showed that patients with COVID-19 infection and invasive mechanical ventilation (IMV) had a higher rate of barotrauma (10% pneumomediastinum and 9% unilateral or bilateral pneumothorax) than patients with acute respiratory distress syndrome and patients without COVID-19 infection [11]. The occurrence of pneumomediastinum and pneumothorax increases in acute medical illnesses as lung failure with a combination of parenchymal injury, as we observed in patients with COVID-19, and severe systemic inflammation is also associated with therapeutic supports with additional positive pressure ventilation as IMV and NIV. Underling comorbidities as COPD may also further increase the risk of these clinical complications.

The common CT manifestations of COVID-19 include bilateral ground-glass opacities (GGO), especially in peripheral, subpleural or posterior areas, with or without consolidations. Chest CT is a diagnostic test to identify and predict COVID-19 in patients with suspected symptoms because of its high sensitivity and high negative predictive value [12,13].

In the context of the COVID-19 infections, the pathogenesis of the pneumomediastinum and pneumothorax is due to alveolar rupture secondary to severe diffuse alveolar and vascular endothelium damage. Episodes of coughing that increase the pressure in the chest, positive pressure ventilation, prone position therapy and steroid therapy may contribute to the development of pneumomediastinum and pneumothorax [14].

Yet, because during pandemic the demand for NIV for patients in the emergency room has been increasing day by day, we suggest considering the risk of pneumothorax and pneumomediastinum in those patients that do not show improvements during NIV. Fast identification of severe clinical complications, such as pneumothorax and pneumomediastinum, is fundamental as far as those of superimposed bacterial infections and/or pulmonary embolism.

In our experience, the occurrence of pneumothorax and pneumomediastinum has been associated with prolonged hospitalization and worse outcomes when overall deaths or deaths due to lung failure are considered.

Although this is a short clinical series, a few reports are present in the Medline regarding pneumothorax and pneumomediastinum with large cohorts. Therefore, our data need to be confirmed on a larger population, but based on this experience, we suggest considering pneumomediastinum and pneumothorax as relevant complications, not only in frail patients that have comorbidities as COPD but also to those that receive NIV support without fast clinical improvements.

## Figures and Tables

**Table 1:** Clinical characteristics of observed patients with pneumomediastinum and pneumothorax.

	Case 1	Case 2	Case 3	Case 4	Case 5	Case 6
Pneumomediastinum	1	1	1	1	1	1
Pneumothorax	0	0	0	0	0	1
Age	63	59	68	78	20	76
Smoking	0	0	0	1	0	0
Males	1	1	1	1	1	1
Diabetes	0	0	0	1	0	0
Coronary disease	0	0	0	1	0	0
Hypertension	0	0	1	1	0	0
COPD	0	0	0	0	0	1
NIPVV	1	1	1	1	0	0
Hospitalization length (days)	33	30	51	40	27	41
Overlapping bacterial pneumonia	0	0	1	1	0	1
Death for non-lung failure	0	0	0	0	0	0
Death for lung failure	0	0	0	1	0	0

## Data Availability

Data is contained within the article.
